# Aster-B–dependent estradiol synthesis protects female mice from diet-induced obesity

**DOI:** 10.1172/JCI173002

**Published:** 2024-01-04

**Authors:** Xu Xiao, John P. Kennelly, An-Chieh Feng, Lijing Cheng, Beatriz Romartinez-Alonso, Alexander Bedard, Yajing Gao, Liujuan Cui, Stephen G. Young, John W.R. Schwabe, Peter Tontonoz

**Affiliations:** 1 Department of Pathology and Laboratory Medicine,; 2 Department of Biological Chemistry,; 3 Department of Microbiology, Immunology & Molecular Genetics, UCLA, Los Angeles, California, USA.; 4 Institute of Structural and Chemical Biology, Department of Molecular and Cell Biology, University of Leicester, Leicester, United Kingdom.; 5 Department of Medicine and Human Genetics, UCLA, Los Angeles, California, USA.

**Keywords:** Metabolism, Adipose tissue, Cholesterol

## Abstract

Aster proteins mediate the nonvesicular transport of cholesterol from the plasma membrane (PM) to the endoplasmic reticulum (ER). However, the importance of nonvesicular sterol movement for physiology and pathophysiology in various tissues is incompletely understood. Here we show that loss of Aster-B leads to diet-induced obesity in female but not in male mice, and that this sex difference is abolished by ovariectomy. We further demonstrate that Aster-B deficiency impairs nonvesicular cholesterol transport from the PM to the ER in ovaries in vivo, leading to hypogonadism and reduced estradiol synthesis. Female Aster-B–deficient mice exhibit reduced locomotor activity and energy expenditure, consistent with established effects of estrogens on systemic metabolism. Administration of exogenous estradiol ameliorates the diet-induced obesity phenotype of Aster-B–deficient female mice. These findings highlight the key role of Aster-B–dependent nonvesicular cholesterol transport in regulating estradiol production and protecting females from obesity.

## Abstract 2

<p>Aster-B-dependent nonvesicular cholesterol transport has a role in regulating estradiol production and protecting females from obesity.</p>

## Graphical abstract

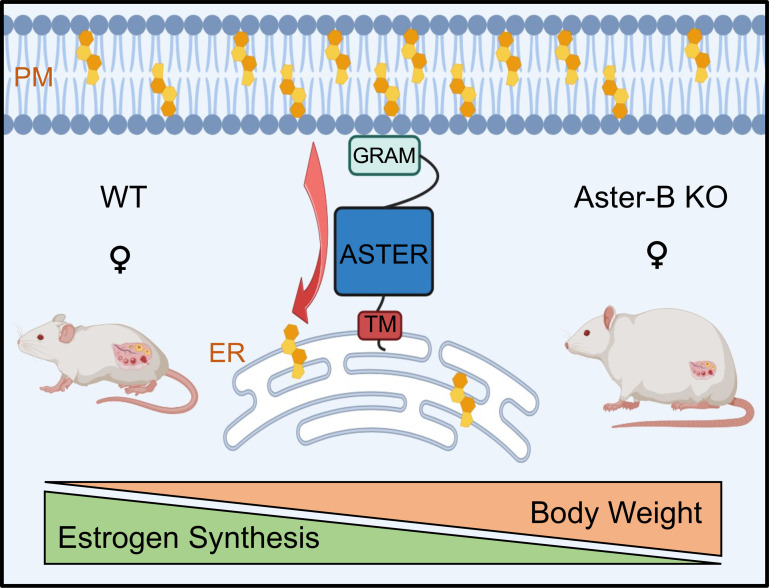

## Introduction

Cholesterol is an integral lipid in mammalian cells, residing primarily in the plasma membrane (PM) (1). Cholesterol must be transported to the endoplasmic reticulum (ER) for use in anabolic processes such as cholesterol esterification, oxysterol production, bile acid synthesis, and steroidogenesis. The Aster family of proteins, including Aster-A, -B, and -C (encoded by *Gramd1a, Gramd1b,* and *Gramd1c* in mice), mediates nonvesicular cholesterol transport from the PM to the ER (2–6). Aster proteins contain a single-pass transmembrane domain that localizes to the ER, an N-terminal GRAM domain that associates with PMs enriched with phosphatidylserine and accessible cholesterol, and a central ASTER domain that mediates cholesterol transport (2).

The 3 Aster proteins are expressed in a tissue-specific pattern in mammals. Prior work showed that Aster-A and -C are highly expressed in hepatocytes. Loss of Aster proteins in the mouse liver impairs cholesterol movement through the liver, leading to decreased cholesterol esterification and bile acid synthesis, elevated plasma cholesterol levels, and cholesterol accumulation in peripheral tissues (7). Aster-B is highly expressed in the adrenal cortex, where its ability to transport HDL cholesterol to the ER is critical for cholesteryl ester (CE) storage and corticosteroid production (2). The role of Aster-B in other steroidogenic tissues remains to be established.

Cholesterol-derived estrogens are crucial steroid hormones that regulate a wide range of physiological processes in women, including bone metabolism, cardiovascular health, cognitive function, and metabolic health (8). Estrogen deficiency has been linked to an increased risk of obesity and metabolic disorders in women (9). Aromatase, encoded by the *Cyp19* gene, catalyzes the final step in the biosynthesis of endogenous estrogens. Mice lacking aromatase function have been shown to accumulate more abdominal adipose and have elevated insulin levels (10). Similarly, in humans, impaired estrogen production during the menopausal transition has been associated with a doubling in the rate of abdominal fat accumulation and worsened cardiometabolic risk factors, including dyslipidemia (8, 11). Interestingly, Aster-B is highly expressed in the ovary, the primary organ responsible for estrogen production in female mice (2). However, it is unknown whether Aster-B–mediated nonvesicular cholesterol transport plays a role in estrogen synthesis, and the potential pathological consequences of the disruption of gonadal nonvesicular cholesterol transport pathways have not been investigated.

In this study, we demonstrated that Aster-B function plays a crucial role in protecting female mice from obesity by facilitating ovarian estradiol synthesis. We identified an isoform of Aster-B carrying an extended N-terminal domain that has enhanced affinity for PM binding and is abundantly expressed in the ovary. Loss of Aster-B function leads to a defect in ovarian PM-to-ER cholesterol transport, causing ovarian hypoplasia and impaired estradiol synthesis. Female Aster-B–deficient mice are vulnerable to diet-induced obesity and hypercholesterolemia to a comparable degree as ovariectomized mice. The accelerated body fat mass gain in female Aster-B–deficient mice is due primarily to lower energy expenditure and locomotor activity. Administration of exogenous estradiol partially rescues the diet-induced obesity in Aster-B–deficient female mice. These data identify a critical role for the Aster-B–mediated nonvesicular cholesterol transport pathway in metabolic homeostasis and protection from diet-induced obesity.

## Results

### Aster-B expression protects female mice from diet-induced obesity.

**Figure 1:**
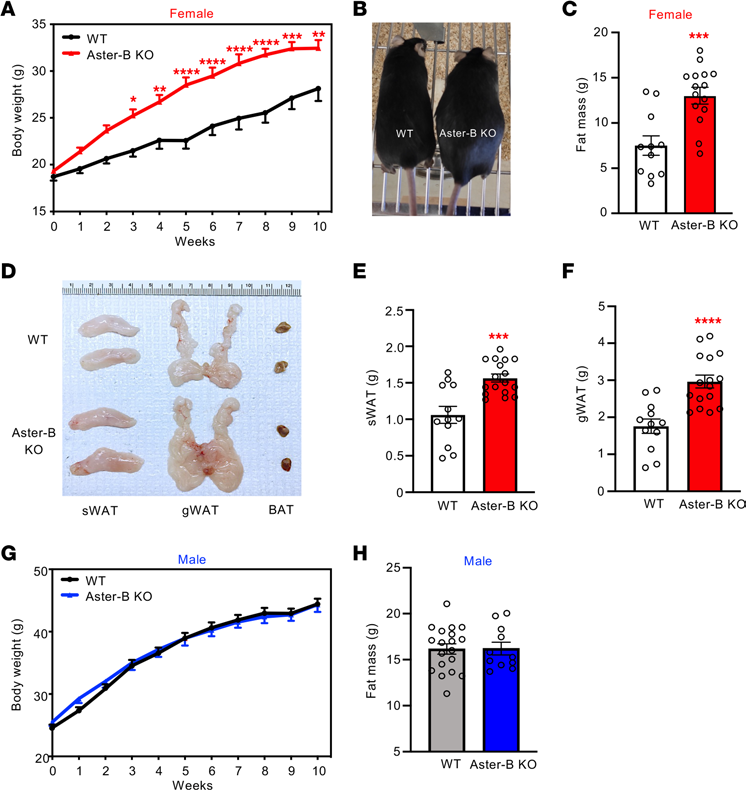
Aster-B expression protects female mice from diet-induced obesity. (**A**) Growth curves for female WT and Aster-B–KO mice fed a WD from 8 weeks of age; *n* = 11 WT and 15 Aster-B–KO mice. (**B**) Representative images of the mice after 10 weeks of WD feeding. (**C**) Body fat mass from female WT and Aster-B–KO mice measured by MRI after 10 weeks of WD feeding; *n* = 11 WT and 15 Aster-B–KO mice. (**D**) Gross appearance of sWAT and gWAT from representative female WT and Aster-B–KO mice after 10 weeks of WD feeding. (**E** and **F**) Weight of sWAT (**E**) and gWAT (**F**) from female WT and Aster-B–KO mice after 10 weeks of WD feeding. *n* = 12 WT and 17 Aster-B–KO mice for sWAT. *n* = 12 WT and 16 Aster-B–KO mice for gWAT (**G**) Growth curves for male mice fed a WD from 8 weeks of age; *n* = 14 WT and 10 Aster-B–KO mice. (**H**) Body fat mass of male mice measured by MRI after 10 weeks of WD feeding. *n* = 19 WT and 11 Aster-B–KO mice. All data are presented as mean ± SEM. *P* values were determined by 2-way ANOVA (**A** and **G**) or 2-sided Student’s *t* test (**C**, **E**, **F**, and **H**). *P < 0.05, **P < 0.01, ***P < 0.001, ****P < 0.0001.

Global Aster-B–KO mice were born at Mendelian ratios without obvious evidence of a generalized developmental growth defect (Supplemental Figure 1, A–C; supplemental material available online with this article; https://doi.org/10.1172/JCI173002DS1). Their body weight was comparable to that of WT controls, regardless of age and sex, when fed a chow diet (Supplemental Figure 1, D and E). To determine the effect of loss of Aster-B function on the progress of obesity, we challenged mice with a western diet (WD) enriched in fat and cholesterol. Female Aster-B–KO mice exhibited accelerated body weight accretion compared with female WT mice, and this trend continued throughout the 10-week study (Figure 1A). Visual inspection revealed that female Aster-B–KO mice had higher adiposity compared with WT mice (Figure 1B). Consistent with these findings, MRI analysis revealed that Aster-B–KO mice had almost twice the amount of fat mass as WT mice, averaging 13 grams compared to 7.4 grams (Figure 1C). The subcutaneous white adipose tissue (sWAT) and gonadal white adipose tissue (gWAT) were expanded in female Aster-B KO compared with WT mice (Figure 1D). We confirmed this by quantifying the weight of sWAT and gWAT (Figure 1, E and F). We found no difference in body weight or fat mass between male Aster-B KO and WT control mice after WD feeding (Figure 1, G and H). Collectively, these data demonstrate that Aster-B expression protects female, but not male, mice from diet-induced obesity.

### Ovarian Aster-B is recruited to the PM by cholesterol loading and transfers cholesterol to the ER.

**Figure 2:**
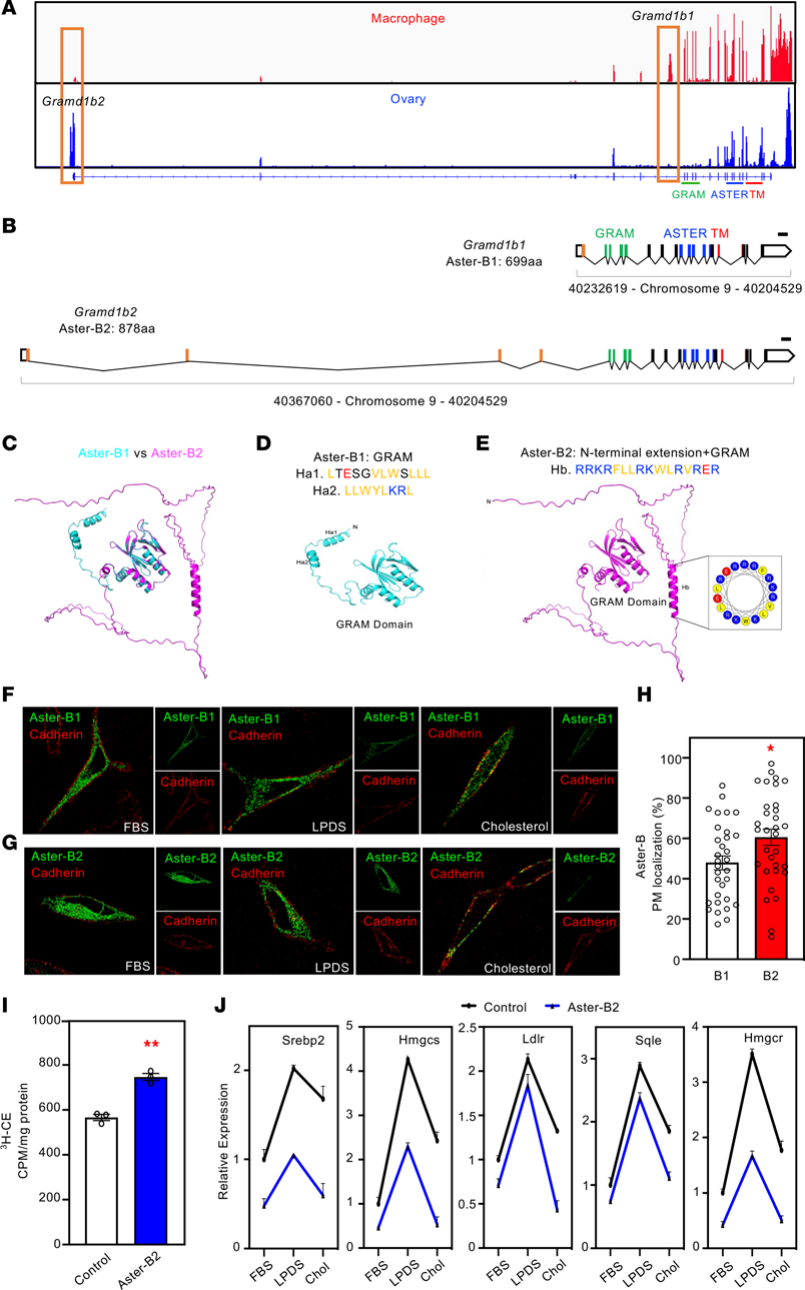
Ovarian Aster-B is recruited to the PM by cholesterol loading. (**A**) RNA-Seq expression of *Gramd1b* in adult C57BL/6J mouse bone marrow-derived macrophages treated with LXR agonist GW3965 and in adult C57BL/6J mouse ovaries (8 weeks of age). Data was reanalyzed from published RNA-Seq data set and visualized as IGV tracks (GEO accession numbers: macrophage: GSE193118; ovary: GSM900183). Exons that correspond to the first exon of *Gramd1b1* and *Gramd1b2* are depicted in organ box, respectively. (**B**) Schematic diagram of 2 different isoforms of Aster-B. Coding exons that are differentially expressed between Aster-B1 and -B2 are depicted in orange. Exons that correspond to the GRAM domains, ASTER domains, and transmembrane (TM) domains are depicted in green, blue, and red respectively. (**C**–**E**) Aster-B1 and B2 are aligned based on the GRAM domain that has the same predicted structure in both proteins (**C**). Alphafold2 structure prediction of GRAM domain of Aster-B1 (**D**), GRAM domain of Aster-B2 (**E**), and helical composition analysis of Hb by HeliQuest (**E**). Nonpolar residues are colored in yellow, positively charged residues are colored in blue, and negatively charged residues are shown in red. (**F** and **G**) Aster-B1 (**F**) and Aster-B2 (**G**) were imaged by confocal microscopy in 10% FBS (left), 1% LPDS (middle), or following 100 μM cholesterol: methyl-b-cyclo-dextrin complexes loading for 1 hour (right). Green, HA tagged Aster-B; red, pan-cadherin. (**H**) Quantification of Aster-B colocalization with PM. *n* = 32 WT and 32 Aster-B–KO mice. (**I**) ^3^H-CE formation in GFP-control and Aster-B2 overexpressed in Aster-A/B/C triple–KO cells. (**J**) Expression levels of the indicated genes in GFP-control and Aster-B2 overexpressed in Aster-A/B/C triple–KO cells that had been cultured with 10% FBS or 1% LPDS for 16 hours. Cholesterol loading was performed by using 10 μM cholesterol: methyl-b-cyclodextrin complexes for 4 hours (Chol). All data are presented as mean ± SEM. *P* values were determined by 2-sided Student’s *t* test (**I**). *P < 0.05,**P < 0.01.

We identified a previously undescribed isoform of Aster-B that is highly expressed in the mouse ovary and adrenal cortex (Supplemental Figure 2A). This isoform has an extended N-terminal region that precedes the GRAM domain (Figure 2A). To differentiate it from the originally characterized shorter isoform in macrophage (2), we refer to this new isoform as Aster-B2, and to the original isoform as Aster-B1. The promoter region for *Gramd1b2* is located upstream and includes 4 additional exons encoding the N-terminal region of the protein, while the first exon expressed in *Gramd1b1* is skipped (Figure 2B and Supplemental Figure 2, B and C).

Both Aster-B1 and B2 are predicted by Alphafold2 to have disordered N-terminal regions (49 amino acids in B1 and 229 in B2). These regions have a very different amino acid composition in the 2 proteins (Supplemental Figure 2D). The protein levels of both isoforms are not influenced by the cholesterol content in the culture media (Supplemental Figure 2E). In both proteins, there are helical elements within this disordered region (Figure 2C). In B1 there are 2 predicted helices that are both largely nonpolar in character (Figure 2D). In B2 a single, longer helix is predicted that is mostly positively charged, but contains some nonpolar residues (Figure 2E). The analysis of this helix by HeliQuest shows a pattern of positively charged residues combined with exposed hydrophobic residues on one side of the helix (Figure 2E). We propose that this helix may promote interaction of Aster-B2 with the PM. Immunofluorescence microscopy experiments showed that, similar to Aster-B1, Aster-B2 was distributed throughout the ER under basal culture conditions. Upon cholesterol loading, Aster-B2 displayed a more pronounced recruitment to the PM (Figure 2F–H). However, Aster-B2 lacking this helix was still robustly recruited to the PM by cholesterol, suggesting that other regions of the amino-terminal (N-terminal) region of Aster-B2 likely contribute directly or indirectly to stronger membrane association (Supplemental Figure 3, A and B). The particularly basic character of the amino terminus of Aster-B2 may be an important factor (Supplemental Figure 3C). These findings collectively suggest that the stronger association of Aster-B2 with the PM compared with Aster-B1 may facilitate cholesterol transport from the PM to ER in steroidogenic tissues.

To directly test if Aster-B2 could transfer cholesterol from PM to ER, we tracked the movement of radio-labeled cholesterol in cells. We overexpressed Aster-B2 in an Aster-A/B/C triple–KO preBAT cell line to bypass the influence of endogenous Asters. KO cells expressing Aster-B2 showed increased accumulation of CE when exposed to ^3^H-cholesterol, indicating that Aster-B2 effectively promoted cholesterol transport to the ER (Figure 2I). The excess cholesterol within the ER also suppressed endogenous cholesterol synthesis. Aster-B2–expressing cells displayed lower SREBP2 pathway gene expression compared with control cells under basal conditions (Figure 2J, FBS). Depletion of cellular cholesterol upregulated SREBP2 pathway activity in control cells; however, this effect was blunted in Aster-B2–expressing cells (Figure 2J, LPDS). Furthermore, reintroducing cholesterol into the cells promoted more effective SREBP2 suppression in Aster-B2–expressing cells (Figure 2J, cholesterol). Immunofluorescence microscopy showed that Aster-B2 primarily colocalized with ER markers, consistent with prior published work on Aster-B1 (2) (Supplemental Figure 3, D–F). These findings collectively provide compelling evidence that Aster-B2 functions similarly to other Asters in transporting cholesterol from the PM to the ER within cells. Note, the ability of Aster-B2 to increase ER cholesterol and suppress SREBP2 strongly argues against a potential role for Asters in moving cholesterol downstream from ER to mitochondria.

### Estradiol synthesis is impaired in Aster-B–KO mice.

**Figure 3:**
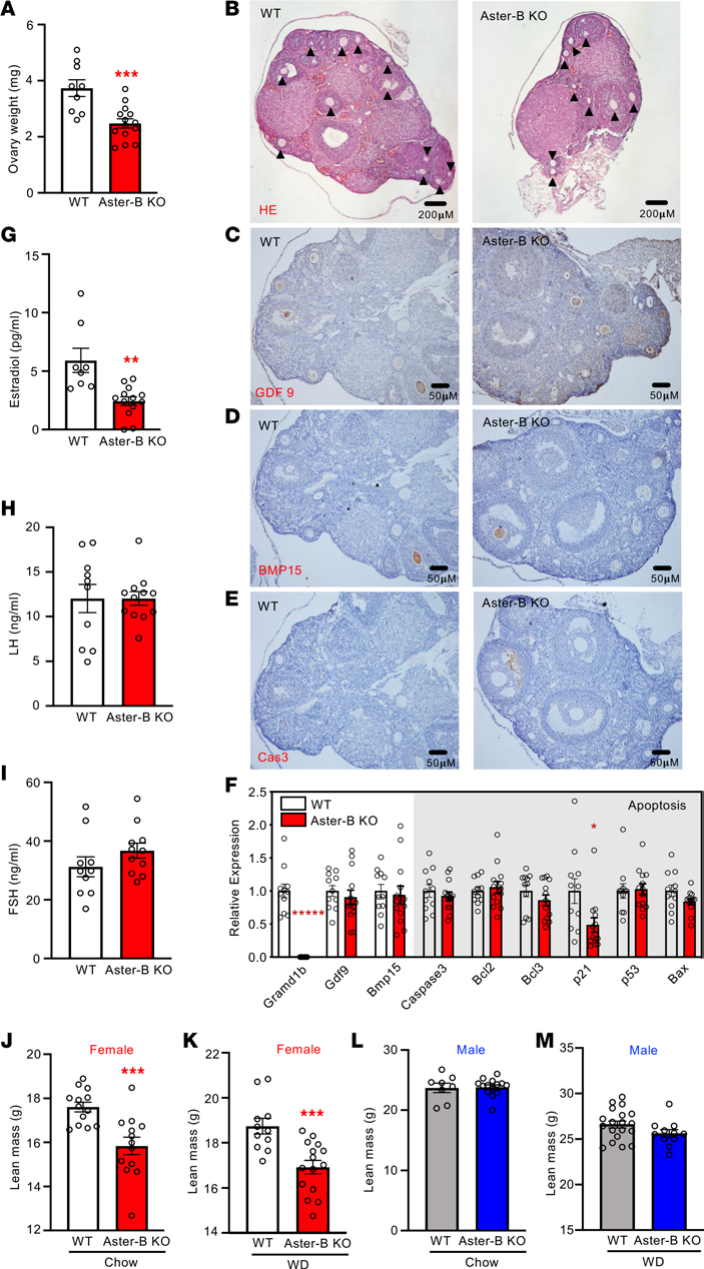
Estradiol synthesis is impaired in Aster-B–KO mice. (**A**) Ovary weights from female WT and Aster-B–KO mice after 10 weeks of WD feeding; *n* = 9 WT and 14 Aster-B–KO mice. (**B**–**E**) Representative stained sections for H&E (**B**), GDP9(**C**), BMP15 (**D**), and Cleaved Caspase3 (**E**) of ovaries from female WT and Aster-B–KO mice. Oocytes in the H&E staining are indicated by the arrow (12 in WT, 9 in Aster-B KO). (**F**) Expression levels of the indicated genes in the ovaries of WT and Aster-B–KO mice. *n* = 11 WT and 13 Aster-B–KO mice. (**G**–**I**) Plasma estradiol (**G**), LH (**H**) and FSH (**I**) from female WT and Aster-B–KO mice. *n* = 8 WT and 13 Aster-B–KO mice for estradiol; *n* = 10 WT and 12 Aster-B–KO mice for LH; *n* = 10 WT and 11 Aster-B–KO mice for FSH. (**J** and **K**) Body lean mass from female mice measured by MRI in chow diet (**J**) or after 10 weeks of WD feeding (**K**). *n* = 13 WT and 13 Aster-B–KO mice for chow diet; *n* = 11 WT and 15 Aster-B–KO mice for WD. (**L** and **M**) Body lean mass from male mice measured by MRI on chow diet (**L**) or after 10 weeks of WD feeding (**M**). *n* = 8 WT and 14 Aster-B–KO mice for chow diet; *n* = 9 WT and 11 Aster-B–KO mice for WD. All data are presented as mean ± SEM. *P* values were determined by 2-sided Student’s *t* test (**A**, **F**, **G**, **H**, **I**, **J**, **K**, **L** and **M**). *P < 0.05, **P < 0.01, ***P < 0.001, *****P < 0.00001

The dramatic female-specific phenotype of Aster-B–KO mice prompted us to investigate ovarian function in these mice. We found that ovaries from female Aster-B–KO mice weighed less than WT control ovaries (Figure 3A). Accordingly, histological analysis demonstrated that the ovaries of female Aster-B–KO mice were smaller in size compared with WT mice (Figure 3B). We also observed a slight reduction in the number of oocytes in the ovaries of Aster-B–KO mice (Figure 3B, arrows). We further analyzed the viability of oocytes by evaluating markers of oocyte health, including GDP9 and BMP15. We found that the ovarian protein and mRNA expression of GDF9 and BMP15 were comparable between Aster-B KO and WT mice by histology and qPCR (Figure 3, C, D, and F). We further found that the apoptosis maker cleaved caspase 3 was elevated in Aster-B KO compared with WT mice (Figure 3E). Accordingly, the ovarian mRNA level of p21, an inhibitor of apoptosis, was decreased (Figure 3F). These results suggested that increased cell death may contribute to hypoplasia and in Aster-B–KO ovaries. To determine if these changes affected fertility, we performed a fertility rate analysis. We found no fertility difference between female Aster-B KO and WT mice at 10 weeks of age (Supplemental Figure 4, A and B). Thus, Aster-B function is not essential for fertility, at least at the ages studied here.

As a major organ for estrogen secretion in females, the ovary plays a critical role in protecting against obesity and metabolic dysfunction before menopause. The fact that Aster-B–KO mice gained more body weight after WD feeding suggested that estradiol synthesis was impaired by loss of Aster-B–mediated cholesterol transport in the ovary. Indeed, the level of estradiol, the major estrogen in plasma, was dramatically lower in female Aster-B KO compared with WT mice (Figure 3G). Luteinizing hormone (LH) is a critical regulator of gonadal function, working synergistically with follicle-stimulating hormone (FSH) to stimulate follicular growth, ovulation, and estrogen synthesis. To determine whether the ovarian hypoplasia and reduced follicle formation in Aster-B–KO mice was due to an upstream hormonal defect, we assessed the levels of LH and FSH. The results showed that the levels of LH and FSH were comparable between groups, indicating that the observed ovarian phenotype was not due to changes in upstream hormones (Figure 3, H and I).

Estrogens are known to correlate positively with muscle and bone mass, and postmenopausal women often experience muscle loss and osteoporosis due to decreased estrogen levels (12, 13). Interestingly, we observed that female Aster-B–KO mice had a lower lean mass on both chow and WD –– a difference not observed in male mice (Figure 3, J–M). This finding suggested that the defect in estradiol synthesis accounts for the lower lean body mass observed in female Aster-B–KO mice. We conclude from these studies that Aster-B plays a crucial role in cholesterol transport from the PM to ER, ultimately affecting estradiol synthesis. Loss of Aster-B results in female mice gaining more weight on WD and having lower lean mass than controls.

### Loss of Aster-B causes hypercholesterolemia in female mice.

**Figure 4:**
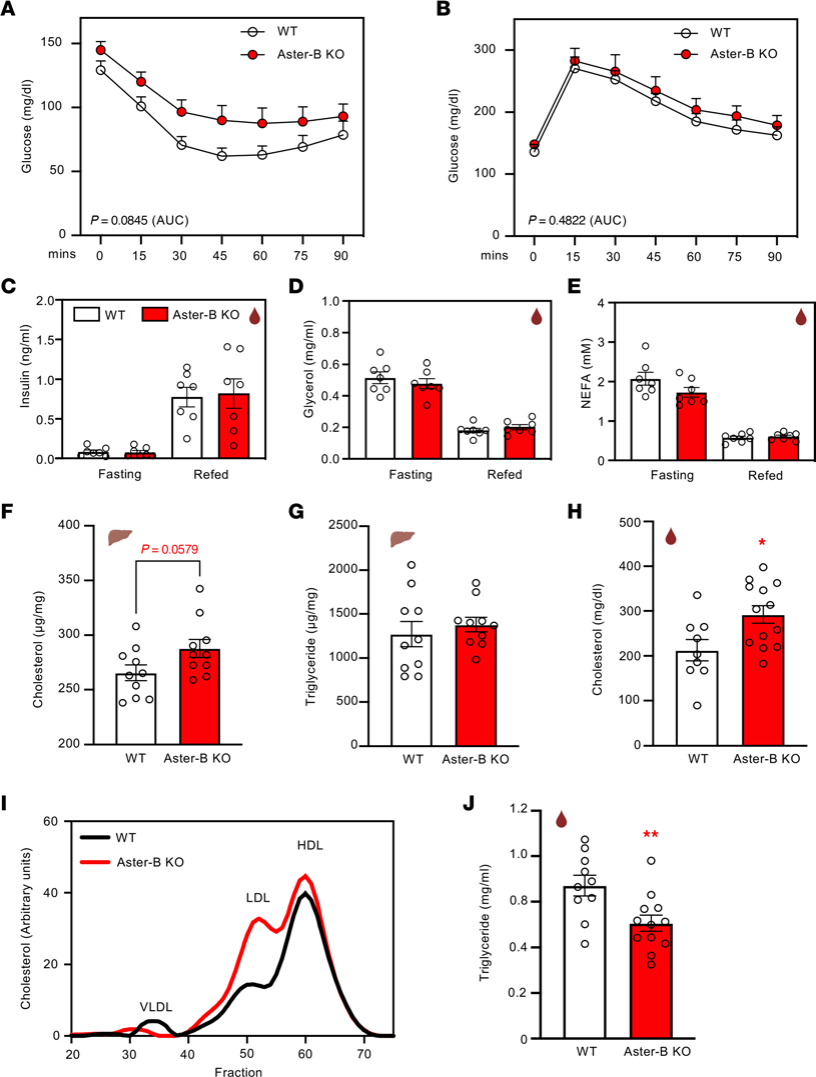
Loss of Aster-B causes hypercholesterolemia in female mice. (**A**) Intraperitoneal insulin tolerance test (1 U kg−1) administered after 10 weeks of WD feeding; *n* = 6 WT and 10 Aster-B–KO mice. (**B**) Intraperitoneal glucose tolerance test (1 mg kg−1) administered after 10 weeks of WD feeding; *n* = 7 WT and 10 Aster-B–KO mice. (**C**–**E**) Plasma insulin, glycerol, and NEFA in female WT and Aster-B–KO mice after 24 hours fasting and refeeding for 2 hours; *n* = 7 WT and 7 Aster-B–KO mice. (**F** and **G**) Total liver cholesterol (**F**) and triglyceride (**G**) in female WT and Aster-B–KO mice after 10 weeks of WD feeding. *n* = 10 WT and 10 Aster-B–KO mice. (**H**) Plasma total cholesterol in female WT and Aster-B–KO mice after 10 weeks of WD feeding; *n* = 9 WT and 13 Aster-B–KO mice. (**I**) Cholesterol content of plasma lipoproteins fractionated from female WT and Aster-B–KO mice after 10 weeks of WD feeding by FPLC. (**J**) Plasma triglyceride in female mice after 10 weeks of WD feeding; *n* = 10 WT and 12 Aster-B–KO mice. All data are presented as mean ± SEM. *P* values were determined by 2-tailed *t* test of the AUC (**A** and **B**), 1-way ANOVA (**C**–**E**) or 2-sided Student’s *t* test (**F**, **G**, **H**, and **J**). *P < 0.05, **P < 0.01.

To evaluate the metabolic fitness of Aster-B–KO mice, we assessed glucose and insulin tolerance after 10 weeks on a WD diet. Female Aster-B–KO mice had a trend toward impaired insulin tolerance, but not glucose clearance (Figure 4, A and B). Plasma levels of insulin, glycerol, and nonesterified fatty acids (NEFA) were comparable between female Aster-B KO and WT mice after 24 hours of fasting and 2 hours of refeeding, respectively (Figure 4, C–E). The increased adiposity in the WD-fed Aster-B–KO mice was also associated with a trend toward higher total cholesterol in the liver, while levels of hepatic triglycerides were similar (Figure 4, F and G). Plasma cholesterol was higher in female Aster-B–KO mice (Figure 4H). This was attributed to increased amounts of both LDL and HDL cholesterol, as assessed by fast protein liquid chromatography (FPLC) analysis (Figure 4I). Plasma triglyceride levels were lower in female Aster-B–KO mice (Figure 4J). Consistent with the lack of body weight difference between male Aster-B KO and WT mice, we did not detect differences in liver or plasma lipids between these groups (Supplemental Figure 5, A–D). These data demonstrate that Aster-B is important for the maintenance of metabolic homeostasis and that loss of Aster-B function leads to hypercholesterolemia in female mice.

### Female Aster-B–KO mice have decreased energy expenditure.

**Figure 5:**
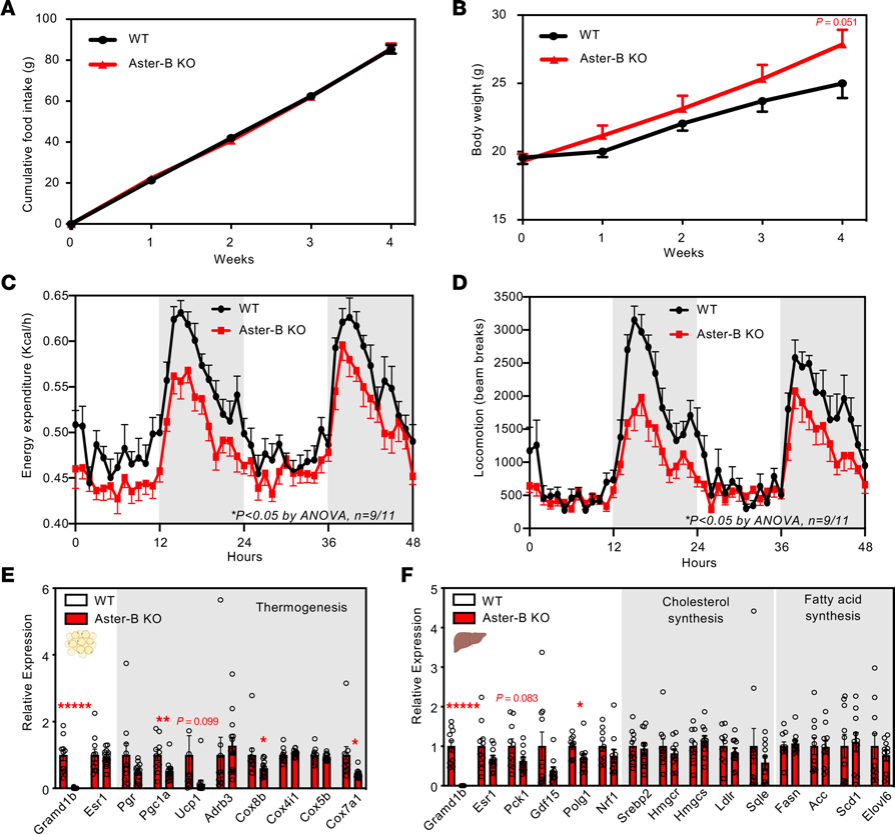
Female Aster-B–KO mice have decreased energy expenditure. (**A**) Cumulative food consumed per mouse from female WT versus Aster-B–KO mice fed with WD for 4 weeks; *n* = 10 WT and 12 Aster-B–KO mice. (**B**) Body weight measures from (A); *n* = 10 WT and 12 Aster-B–KO mice. (**C**) Energy expenditure measured by indirect calorimetry in mice after 4 weeks on WD; *n* = 9 WT and 11 Aster-B–KO mice. (**D**) Locomotor activity as measured when a mouse crossed multiple infrared beams during the calorimetry experiment; *n* = 9 WT and 11 Aster-B–KO mice. (**E** and **F**) Expression levels of the indicated genes in the sWAT (**E**) and liver (**F**) of WT and Aster-B–KO mice. *n* = 10 WT and 14 Aster-B–KO mice for sWAT; *n* = 10 WT and 10 Aster-B–KO mice for liver. All data are presented as mean ± SEM. *P* values were determined by 2-way ANOVA (**A**–**D**) or 2-sided Student’s t-test (**E** and **F**). *P < 0.05, **P < 0.01, *****P < 0.00001.

We next sought to investigate the mechanisms underlying the obesity phenotype of female Aster-B–KO mice. As obesity results from an imbalance between calorie intake and output, we analyzed food intake and body weight of singly housed mice (WT, *n* = 10; Aster-B KO, *n* = 12) over 4 weeks. Despite similar food intake between groups, female but not male Aster-B–deficient mice gained more body weight than WT controls (Figure 5, A and B and Supplemental Figure 6, A and B). These data suggested that the obesity phenotype of female Aster-B–KO mice is not due to increased food intake. Estrogens have been shown to regulate energy homeostasis in white adipose tissue (WAT) and brown adipose tissue (BAT) (14, 15). We hypothesized that the susceptibility to diet-induced gain in Aster-B–KO mice could involve changes in energy expenditure. To test this, we performed indirect calorimetry on the female WT and Aster-B–KO mice using metabolic chambers. Female Aster-B–KO mice had decreased energy expenditure (*P* < 0.05 by ANOVA) when either the overall mean total body mass or mean lean body mass of each group was used as the covariate (Figure 5C). In addition to stimulating energy expenditure, estrogens have been shown to increase locomotor activity in mice through estrogen receptor α (16). We found that loss of Aster-B also decreased locomotor activity (Figure 5D), which could further contribute to the decreased energy expenditure observed in female Aster-B–KO mice. We did not observe changes in the respiratory exchange ratio (RER), suggesting that substrate utilization was comparable between groups (Supplemental Figure 6C). Collectively, these studies demonstrate that the increased body weight of female Aster-B–KO mice is due to decreased estrogen-induced energy expenditure.

We assessed systemic effects resulting from the loss of Aster-B-dependent estradiol synthesis by measuring estrogen-responsive transcripts in adipose tissue and liver. Notably, while *Esr1* and *Pgr* expression were not different, we observed a reduction in the expression of thermogenesis-related genes, including *Pgc1a*, *Cox8b*, and *Cox7a1*, in the sWAT of female Aster-B KO compared with WT mice (Figure 5E). This finding aligns with the energy expenditure phenotype described in Figure 5C for female Aster-B–KO mice. Additionally, in the liver, our analysis revealed a trend toward lower expression of the estrogen-responsive genes *Pck1* and *Polg1* (14). There were no alterations in the expression of genes associated with cholesterol and fatty acid synthesis in Aster-B–deficient mice (Figure 5F).

### Female Aster-B–KO mice mimic ovariectomized mice.

**Figure 6:**
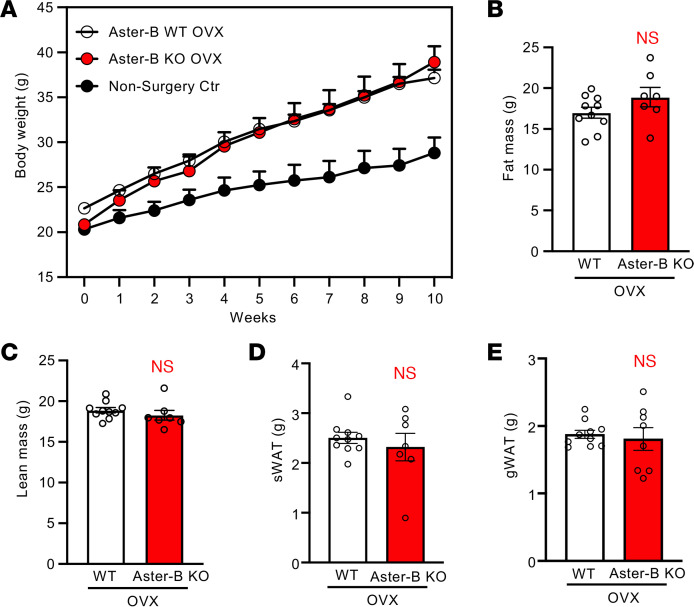
Female Aster-B–KO mice mimic ovariectomized mice. (**A**) Growth curve for nonsurgery control and ovariectomized WT or Aster-B–KO mice fed a WD from 8 weeks of age; *n* = 10, 8, and 8, respectively. (**B** and **C**) Body fat mass (**B**) and lean mass (**C**) from ovariectomized female WT and Aster-B–KO mice measured by MRI after 10 weeks of WD feeding; *n* = 10 and 7, respectively. (**D** and **E**) Weight of sWAT (**D**) and gWAT (**E**) from ovariectomized female mice after 10 weeks of WD feeding; *n* = 10 and 7, respectively for sWAT; *n* = 10 and 8, respectively for gWAT. All data are presented as mean ± SEM. *P* values were determined by 2-way ANOVA (**A**) or 2-sided Student’s *t* test (**B**–**E**).

To further verify that the ovary was the primary organ responsible for the defects in female Aster-B–KO mice, we performed ovariectomy on WT and Aster-B–KO mice (Supplemental Figure 7A). As expected, ovariectomized WT mice gained more body weight than nonovariectomized mice after WD feeding (Figure 6A and Supplemental Figure 7B). However, the body mass of the Aster-B KO and WT groups was indistinguishable throughout the study (Figure 6A and Supplemental Figure 7B). Similarly, MRI analysis revealed that the differences in body fat mass, lean mass, and sWAT/gWAT mass between WT and Aster-B–KO mice were blunted after ovariectomy (Figure 6, B–E). Taken together, these results strongly suggest that the ovary is the primary site of the defect in female Aster-B–KO mice leading to the obesity phenotype.

### Exogenous estradiol ameliorates diet-induced obesity in female Aster-B–KO mice.

**Figure 7:**
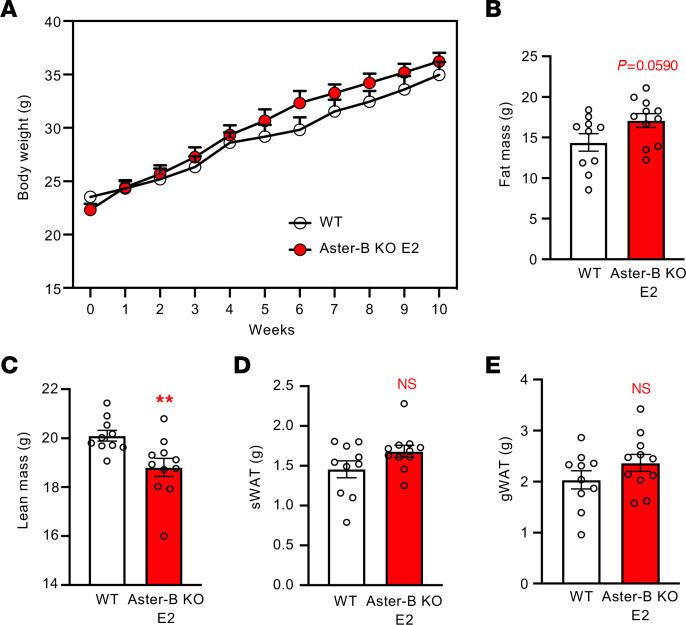
Exogenous estradiol ameliorates diet-induced obesity in female Aster-B–KO mice. (**A**) Growth curve for WT versus E2-treated Aster-B–KO mice fed a WD from 8 weeks of age. *n* = 10 and 11, respectively. (**B** and **C**) Body fat mass (**B**) and lean mass (**C**) from WT and E2-treated Aster-B–KO mice measured by MRI after 10 weeks of WD feeding; *n* = 10 and 11, respectively. (**D** and **E**) Weight of sWAT (**D**) and gWAT (**E**) from WT and E2-treated Aster-B–KO mice after 10 weeks of WD feeding; *n* = 10 and 11, respectively. All data are presented as mean ± SEM. *P* values were determined by 2-way ANOVA (**A**) or 2-sided Student’s *t* test (**B**–**E**). **P < 0.01

To test whether Aster-B was acting through estradiol to protect female mice from obesity, we administered 100 ng/mL estradiol to female Aster-B–KO mice in their drinking water during WD feeding. Exogenous estradiol ameliorated the diet-induced body weight gain in Aster-B–KO mice (Figure 7A and Supplemental Figure 7C). MRI analysis showed that the increase in fat mass, but not the reduction in lean mass, was also blunted after estradiol administration (Figure 7, B and C). Consistent with these findings, the weights of sWAT and gWAT were similar between WT and Aster-B–KO mice after estradiol administration (Figure 7, D and E). Together, these results suggest that Aster-B mediated PM-to-ER cholesterol transport is critical for estradiol synthesis in the ovary and that reduced estradiol production in Aster-B–KO mice predisposes to obesity after WD feeding.

## Discussion

Aster proteins mediate nonvesicular cholesterol transport of accessible cholesterol from the PM to ER (2). Loss of Aster function expands the PM accessible cholesterol pool at the expense of the ER, leading to decreased CE synthesis and steroidogenesis in the liver and adrenal glands (2–4, 7). However, the role of Asters in metabolic disease has not been defined. Here we showed that an extended form of Aster-B, with greater PM affinity (Aster-B2), plays a critical role in transporting PM cholesterol to the ER to support estradiol synthesis in the ovary. Reduced estradiol production in female Aster-B–KO mice leads to reduced locomotor activity and energy expenditure. Moreover, female Aster-B–KO mice develop obesity and hypercholesterolemia when fed a WD. These findings define a critical role for nonvesicular cholesterol transport in ovarian function and provide a link between Aster-B function and metabolic disease susceptibility.

We previously reported that loss of Aster-B decreased cortisol synthesis in the adrenal gland (2). Cortisol secretion has been extensively examined in the context of human obesity, although its mechanistic connections are incompletely understood. Of potential relevance to the present work, studies have suggested that higher cortisol levels may promote food intake in humans with obesity (17, 18). However, our data strongly suggest that the obesity phenotype of female Aster-B–KO mice is due to impaired estradiol synthesis, rather than cortisol deficiency. First, Aster-B–KO mice have lower, not higher, levels of cortisol in their plasma. Second, female Aster-B–KO mice do not exhibit increased food intake. Third, male Aster-B–KO mice fed a WD developed obesity at the same rates as WT mice. Finally, exogenous administration of estradiol partially rescued the diet-induced obesity phenotype of female Aster-B–KO mice.

The ovary is the predominant source of estrogen production in females, but estrogens can also be synthesized in nonreproductive tissues including adipose tissue, liver, heart, muscle, bone, and brain (19). We cannot exclude the possibility that Aster-B–mediated cholesterol transport may also play a role in supplying cholesterol for estrogen synthesis in these tissues. However, the fact that ovariectomized WT mice mimic the body weight gain of Aster-B–KO mice on a WD diet suggests that the ovary is the source of the primary defect. Furthermore, the levels of FSH and LH were comparable between WT and Aster-B–KO mice, suggesting that upstream hormone secretion in the pituitary was normal. We also found that apoptosis was elevated in the ovaries of Aster-B KO compared with WT mice (Figure 3E). These results suggest that increased cell death may contribute to impaired estrogen synthesis in Aster-B–KO ovaries.

The primary source of cholesterol for steroid synthesis in rodents is extracellular HDL. Asters mediate the transfer of cholesterol from the HDL receptor at the PM to the ER. Loss of Asters therefore impairs cholesterol flux to the ER, and this reduces substrate availability for steroid synthesis in mitochondria. We did not observe significant colocalization between Aster-B2 and mitochondria. In contrast to Aster-B–KO mice, the adrenal glands of mice lacking the mitochondrial sterol transporter StarD1 display CE accumulation as a consequence of defective mitochondrial cholesterol import. The phenotypic differences between StarD1 KO and Aster-B–KO mice are consistent with a primary role for Asters in PM-to-ER cholesterol transport. StarD1 and perhaps other as yet unidentified factors mediate cholesterol transfer from ER to mitochondria.

Estrogens play important roles in the development and maturation of bone and muscle, as well as in maintaining bone density and muscle strength (20). In humans, menopause leads to a loss of bone and muscle mass and strength (12, 13). We observed that female Aster-B–KO mice have lower lean body mass compared with WT mice, a finding reminiscent of the lower lean body mass observed in Aromatase-KO mice (10). Therefore, it is plausible that bone and muscle mass loss contribute to the lower lean body mass observed in female Aster-B–KO mice. Muscle is well recognized for its role in the regulation of insulin sensitivity. The reduction in lean mass, which includes contributions from muscle tissue, likely contributes to the observed trend of impaired insulin tolerance, as depicted in Figure 4A. A similar observation was found in the Cyp19-KO mice, which had elevated levels of insulin, but not glucose levels, compared with WT mice at 1 year of age (10).

While female Aster-B–KO mice exhibited increased obesity, their plasma triglyceride levels were lower than those found in WT mice. FPLC analysis revealed a notable reduction in very low-density lipoprotein (VLDL) levels in the plasma of female Aster-B–KO mice (Figure 4I). A possible explanation is that VLDL secretion might be impaired in female Aster-B–KO mice compared with WT mice. Impaired VLDL secretion typically results in elevated TG levels in the liver (21–23). However, the TG level was identical in the livers of female Aster-B KO and WT mice. These results suggest that the reduction of plasma triglyceride could be due to in Aster-B function in other tissues. Estrogen administration is known to increase plasma triglyceride levels by elevating large triglyceride-rich VLDL particles in humans (24). This is associated with a decline in hepatic triglyceride lipase following estrogen administration (25). Therefore, the reduction of estrogen could also contribute to the lower plasma triglyceride levels observed in female Aster-B KO compared with WT mice.

Despite the hypoplasia and reduced estradiol production of Aster-B–deficient ovaries, we did not observe obvious developmental defects in female Aster-B–KO mice. It appears that the female Aster-B–KO mice are still capable of synthesizing enough estradiol to maintain their normal development, possibly through other nonreproductive tissues or by increasing endogenous cholesterol synthesis. The expression of markers of oocyte health, like GDF and BMP15, were comparable in the ovary of female Aster-B KO and WT controls (Figure 3, C, D, and F). Female Aster-B–KO mice also had similar fertility ratios as WT mice at 10 weeks of age. However, the ovaries of Aster-B–KO mice had fewer oocytes than WT mice, suggesting that they may become depleted more rapidly with aging. Further studies are needed to test this hypothesis.

In conclusion, our data reveal a critical role for Aster-B–mediated nonvesicular cholesterol transport in supporting ovarian estradiol synthesis in mice. Loss of Aster-B leads to diet-induced obesity by reducing energy expenditure and locomotor activity in female mice. These findings advance our understanding of the physiologic and pathologic roles of Aster-B and may highlight potential opportunities for targeting Aster-B in the diagnosis and treatment of female obesity.

## Methods

### Mice.

Aster-B–KO mice were previously described (2). Briefly, the mice were generated at Mouse Biology Program facility on a C57BL/6N background using the CRISPR/Cas9 strategy. All mice were housed in climate-controlled facilities (22 °C with humidity approximately 50%–65%) with a 12-hour light-dark cycle and under pathogen-free conditions. Experiments were performed on male and female mice. Mice were fasted for 4 hours beginning at either 8 or 9 AM before being killed to collect plasma and tissues.

### Diet feeding.

Mice were fed a chow diet (PicoLab Rodent Diet 20, 5053) or WD (Research Diets, D12079B). For WD studies, dietary challenge was initiated in 8-week-old littermate mice. Body weights were recorded on a weekly basis.

### Histology.

Ovaries were fixed in 4% paraformaldehyde and stored in 75% ethanol before being mounted in paraffin. Sections (10 μm) were cut and stained with H&E, GDF9 (Thermo Fisher Scientific) and BMP19 (Thermo Fisher Scientific) by the UCLA Translational Pathology Core.

### Plasma estradiol, LH, and FSH measurements.

For the hormone measurement, we synchronized estrous cycles of female mice by introducing soiled bedding from a male’s cage into the females’ cage 3 days before collecting blood samples. The plasma estradiol was measured by ELISA assay (Calbiotech, Inc Estradiol (E2) ELISA Kit). The plasma LH and FSH was measured by ELISA assay (Mybiosource, Mouse LH ELISA Kit; Mouse FSH ELISA Kit).

### Glucose tolerance test and insulin tolerance test.

Mice were fasted for 4 hours beginning at 9 AM before the glucose and insulin tolerance tests. Glucose tolerance tests and insulin tolerance tests were determined by administering an intraperitoneal glucose (1 g/kg) or insulin (1 U/kg) at week 9. Blood samples were collected from the tail vein at indicated times. Blood glucose was by measured by Bayer Contour Next Blood Glucose Test Strips.

### Liver and plasma lipid measurements.

Livers were disrupted in homogenization buffer (10 mM Tris-HCl [Bioland], pH 7.4, 150 mM NaCl [Sigma-Aldrich], 1 mM EDTA [Bioland], and protease inhibitors [Thermo Fisher Scientific]) and lipids were extracted from 0.5–1 mg liver protein using the Folch method (26). Liver and plasma cholesterol and triglyceride were measured by colorimetric assay (cholesterol, WAKO; TG, Sekisui; glycerol, Cayman [biomol]; NEFA, Fujifilm, HR Series NEFA-HR). Plasma insulin was measured by Mouse Insulin ELISA Kit (Thermo Fisher Scientific). To resolve lipoprotein classes, plasma samples were injected into a Superose 6 10/300 (GE Healthcare Life Sciences) column attached to ÄKTA Pure FPLC unit (GE Healthcare) and fractions were collected for measurement of cholesterol by colorimetric assay (WAKO).

### Food intake.

Littermate mice were singly housed in standard housing and fed a WD ad libitum. The mass of diet administered and consumed was recorded weekly. We monitored food intake for 4 weeks.

### Metabolic chamber.

The baseline MRI analysis was performed immediately before the calorimetry experiments. Mice were singly housed from the time they were moved to the metabolic chambers until the end of the study (48 hours). Energy expenditure and locomotion activity were measured by indirect calorimetry using the Oxymax Comprehensive Laboratory Animal Monitoring System (Columbus Instruments). Data were analyzed using CalR (https://calrapp.org).

### Ovariectomy.

The ovariectomy protocol was adapted and modified from previous studies (27). Briefly, the hair was shaved from the flank and the area disinfected. The musculature was separated carefully and the ovarian fat pad removed through the incision. The region below the ovary was clamped and sterile thread used to use to delineate the region to be removed. Steps were repeated on the contralateral side. The body wall was sutured with 1 or 2 stitches and the skin closed with wound clips. Baytril was administered to prevent infection.

### Fertility rate analysis.

Female WT and Aster-B–KO mice were singly paired with a male WT mouse from Jackson Laboratories at 10 weeks of age. The number of litters and pups were recorded over a 10-week time period.

### GRAM domain structure prediction.

Protein structure prediction was performed using AlphaFold2, a machine-learning prediction software for protein structure based on sequence and multiple sequence alignment (28). The helix analysis of the predicted helices was made by Heliquest, a software that calculates the physicochemical properties of a helix from the amino acid sequence and composition (29).

### Aster-B localization.

HeLa cells (ATCC, CCL-2) were plated in 24-wells plates with 10,000 cells/well on Poly D-lysine coated sterile German glass coverslips. The cells were transfected with 200 ng of Aster-B by Fugene 6 (Promega) following manufacturer’s protocol. The cells were maintained in DMEM supplemented with 10% FBS overnight. Cells were then either maintained in DMEM supplemented with 10% FBS or DMEM supplemented with 1% lipoprotein-deficient serum (LPDS) and 5 μM simvastatin and 10 μM mevalonate for cholesterol depletion for 16 hours. For cholesterol loading, cells were incubated with 100 μM cholesterol: methyl-b-cyclo-dextrin (randomly methylated, Sigma-Aldrich) complexes for 1 hour before being washed 3 times with PBS and fixed with 4% PFA for 15 minutes. After fixation, cells were washed 3 times with PBS. Cells were incubated with anti-HA (Cell Signaling Technology, 3724S, diluted 1:1,000), Pan-cadherin (Abcam, ab6528, diluted 1:2,000) antibodies overnight before incubation with fluorescently labeled secondary antibodies (goat anti-rabbit IgG H&L Alexa Fluor 488, Invitrogen A11034, diluted 1:1,000; goat anti-rabbit IgG H&L Alexa Fluor 555, Invitrogen A21428, diluted 1:1,000; MitoTracker Green FM, Invitrogen M7514, diluted 1:1,000) for 1 hour at room temperature. Cells were mounted on slides with ProLong Diamond Antifade Mountant with DAPI (Invitrogen). Images were acquired using an Inverted Leica TCS-SP8-SMD Confocal Microscope.

### Western blot.

Aster-A/B/C triple–KO cells were generated by treating immortalized fibroblasts (preadipocytes) derived from Aster A/B/C-floxed mice with Cre virus in vitro before selecting and verifying KO clones. Aster-A/B/C triple–KO cells were infected by lentivirus expressing control GFP or HA tagged Aster-B1 or B2 in 6-well plates with 3,000 cells/well. The cells were maintained in DMEM supplemented with 10% FBS overnight. Cells were then switched to DMEM supplemented with 1% LPDS with 5 μM simvastatin and 10 μM mevalonate for 16 hours to deplete cholesterol. For cholesterol loading, cells were incubated with100 μM cholesterol: methyl-b-cyclo- dextrin complexes for 4 hours before being harvested for immunoblot. Whole cells were lysed using RIPA buffer (Boston BioProducts) supplemented with protease inhibitor (Thermo Fisher Scientific) and phosphatase inhibitor (Thermo Fisher Scientific) cocktail tablets. Protein concentrations were measured using the Bicinchoninic Acid Assay Protein Assay kit (Thermo Fisher Scientific), and Western blots were performed for HA tag (Cell Signaling Technology, 3724S), GFP (Abcam, ab6556), and β-Actin (Sigma, A5316-100UL) followed by secondary antibodies (goat anti-rabbit HRP, Fisher Scientific, 656120; goat anti-mouse HRP, Fisher Scientific, NC9731556) for 1 hour at room temperature. Images were acquired by ImageQuant LAS4000 (GE Healthcare).

### Gene expression analysis.

RNA was extracted from cells or tissues using TRIzol reagent (Invitrogen) for quantitative PCR. Complementary DNA was generated by reverse transcription and quantified using iTaq Universal SYBR Green Supermix (Bio-Rad\) and the QuantStudio 6 Flex 384-well qPCR system (Applied Biosystems). Each gene of interest was normalized to *36b4*. Quantitative PCR primers can be found in Supplemental Table 1.

### ^3^H-CE formation.

Cells were depleted of sterols by incubating in a medium containing 1% LPDS (Sigma-Aldrich), simvastatin (5 μM; Sigma-Aldrich), and mevalonate (10 μM; Sigma-Aldrich) for 16 hours at 37°C. Cells were switched to the same medium for another 4 hours with 0.1μCi ^3^H-cholesterol. Lipids were extracted from the cells by the Folch method. Cholesterol and CE were separated by TLC on silica plates using the solvent system heptane: isopropyl ether: acetic acid (60:40:4). The radioactivity in each lipid fraction was determined by liquid scintillation counting after scraping the relevant bands. ^3^H radioactivity in CEs was normalized per mg protein.

### Image quantification.

Images were processed as follows: Images were reconstructed and up sampled using a sparse deconvolution (Sparse-SIM v1.03) package for MATLAB with the following parameters: sparse iterations = 100, image fidelity = 40, sparsity = 0.4, deblur times = 7, background = Strong Background (High Intensity), Lucy-Richardson algorithm with fourier upsampling. Reconstructed images were further processed with an adaptive median filter in ImageJ. Processed images were quantified in CellProfiler after double-positive Aster-B/Cadherin cells were manually identified. The radial distribution of the Aster signal intensity within the cadherin signal was recorded.

### Statistics.

All data are presented as mean ± SEM and analyzed using Prism (GraphPad v.9). A 2-tailed Student’s *t* test was used for single variable comparison between 2 groups. A 1-way ANOVA with Tukey’s or Dunnett’s posttest was used to compare a single independent variable between multiple groups. A 2-way ANOVA followed by Šidák’s posttest was used to examine interactions between multiple variables. *P* < 0.05 was considered statistically significant.

### Study approval.

All animal experiments were approved by the UCLA Institutional Animal Care and Research Advisory Committee. Experimental mice were sacrificed as specified for histological, serum, lipid, and gene expression analyses.

### Data availability.

See complete unedited blots in the supplemental material. The data that support the findings of this study are available in the Supporting Data Values or from the corresponding author upon request.

## Author contributions

XX designed, performed, and analyzed the results of most of the experiments, assembled the figures, and wrote the manuscript. JPK assisted in the sacrifice of mice, performed the FPLC analysis and edited the manuscript. ACF and L Cheng performed the ovariectomy surgery. BRA and JWRS performed the GRAM domain structure prediction analysis. AB and YG analyzed the ovarian RNA-Seq data and confocal image quantification. L Cui assisted in the confocal imaging. PT and SGY designed and supervised the overall research and wrote the manuscript.

## Supplementary Material

Supplemental data

Unedited blot and gel images

Supporting data values

## Version 1 01/04/2024

In-Press Preview

## Version 2 02/15/2024

Electronic publication
