# Supplementary material for: Aster-B–dependent estradiol synthesis protects female mice from diet-induced obesity
Source: J Clin Invest. 2024 Jan 4;134(4):e173002. doi: 10.1172/JCI173002 (PMC10866650; doi:10.1172/JCI173002)
Supplement: Supplemental data [file jci-134-173002-s111.pdf]

Supplemental Figure 1. (**A and B**) Expression levels of the indicated genes in the ovaries (A) and liver (B) of female WT and Aster-B KO mice. n = 11/13 for ovary; n = 10/10 for liver. (**C**) Representative images of the female mice on chow diet feeding. (**D**) Body weight of female WT and Aster-B-KO mice on chow diet at age of 12 weeks, 20 weeks and 30 weeks; n = 20/31 for 12 weeks; n = 15/16 for 20 weeks; n = 13/13 for 30 weeks. (**E**) Body weight of male WT and Aster-B-KO mice on chow diet at age of 12 weeks, 20 weeks and 30 weeks; n = 16/25 for 12 weeks; n = 11/13 for 20 weeks; n = 16/21 for 30 weeks. All data are presented as mean ± s.e.m. P values were determined by two-sided Student's t-test (A, B, D and E). *****P < 0.00001.

Supplemental Figure 2. (**A**) Relative expression of *Gramd1b* in different tissues from the mouse. n=5. (**B and C**) cDNA sequence of *Gramd1b1*(B) and *Gramd1b2* (C). (**D**) ClustalW alignment of N-terminal region from Aster-B1 and B2. The rest of the proteins share the same amino acid sequence. The position of the predicted helices in Aster-B1 and B2 are highlighted in cyan and magenta, respectively. (**E**) Immunoblot analysis of Aster-B1 and -B2 after vehicle or indicated cholesterol treatment for 4 hours.

Supplemental Figure 3. (**A** and **B**) Aster-B2 (A) and Aster-B2 truncated α-Helix (B) in Hela cells imaged by confocal microscopy in 10% FBS (left), 1% LPDS (middle) or following cholesterol loading for 1 h (right). Green, HA tagged Aster-B. Blue, DIPA. (**C**) Amino acid composition of Aster-B1 and B2 N-terminal region. Non-polar residues in Aster-B1 are highlighted in yellow and positively charged residues are highlighted in blue in Aster-B2. (**D**) Aster-B2, Mito-tracker and KDEL-BFP were imaged by confocal microscopy in 10% FBS (left), 1% LPDS (middle), or following 100 μM cholesterol: methyl-b-cyclo- dextrin complexes loading for 1 h (right).

Supplemental Figure 4. (**A**) Average number of birth pups per female in 10 weeks; n = 6/6. (**B**) Fertility rate analysis for female WT and Aster-B knock-out mice in 10 weeks. All data are presented as mean ± s.e.m. P values were determined by two-sided Student's t-test (A).

Supplemental Figure 5. (**A and B**) Total liver cholesterol (A) and triglyceride (B) in male WT and Aster-B-KO mice after 10 weeks of WD feeding. n=10/10. (**C and D**) Plasma total cholesterol (C) and triglyceride (D) in male WT and Aster-B-KO mice after 10 weeks of WD feeding; n = 18/11 for cholesterol; n=19/11 for triglyceride. All data are presented as mean ± s.e.m. P values were determined by two-sided Student's t-test (A, B, C and D).

Supplemental Figure 6. (**A**) The cumulative food consumed per mouse from male WT versus Aster-B-KO mice fed with WD for 4 weeks of study. n=6/4. (**B**) The body weight measured from (A); n=5/4. (**C**) The Respiratory Exchange Ratio (RER) data from Figure 5C and 5D. n= 10/12. All data are presented as mean ± s.e.m. P values were determined by two-way ANOVA (A, B) or two-sided Student's t-test (C).

Supplemental Figure 7. (**A**) Representative image of mice with or without ovariectomy. (**B**) Normalized body weight gain from Figure 1A, and the ovariectomized WT or Aster-B-KO mice are from Figure 6A. n = 10/8/11/15. (**C**) Normalized body weight gain from Figure 1A and E2 treated Aster-B-KO mice from Figure 7A. The WT group combined the data from Figure 1A and 7A. n = 21/11/15.

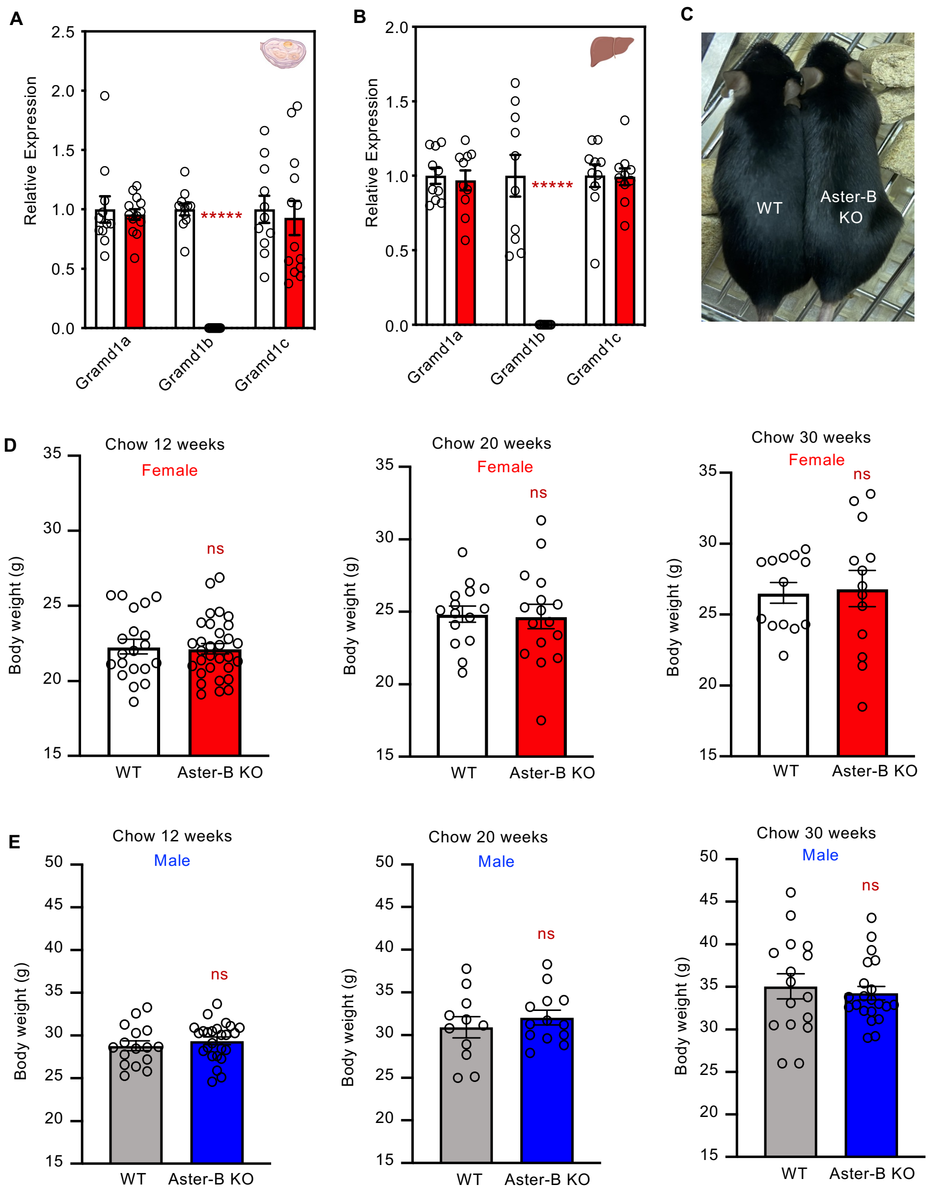
A
B
C
Relative Expression
*****
Gramd1a
Gramd1b
Gramd1c
WT
Aster-B KO
D
Chow 12 weeks
Female
ns
Chow 20 weeks
Chow 30 weeks
Body weight (g)
E
Male

Xiao_Fig S1.

A

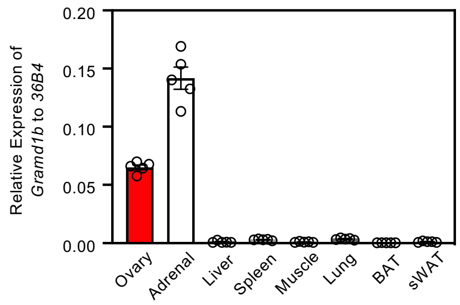

B

Mouse *Gramd1b1* Full length Sequence

Exon 1;

GRAM domain; ASTER domain; TM

atggaatctctgacagaatctggggtcctctggagcctcctgctggaactggacagccagtctctgctgtggtacttgaagaggcttgctgatgcacctgttggtgccgaatgctactgctggcacggatctgagaaaa
tcccagccgtgctaagccccacttacaagcagagaaatgaagacttcagaaagctctttaagcaacttccagacacggagcgcctcattgttgattactcttgtgcactccaaagagacattcttcttcagggccgac
tctacctctcagaaaattggatctgcttctacagcaacatcttccgctgggaaaccctgctgacagtccgtttgaaagatatctgctccatgactaaagagaaaacagctcgcctcattcccaatgccatccaagtctg
cactgattcagaaaagcactttttacttcctttggagcccgggacaggacgtacatgatgatgttccggctctggcagaatgctctccttgaaaagcccctatgtcccaaggagctctggcactttgtccaccagtgct
acgggaacgagctgggcctaaccagtgatgatgaggactacgtgcctccagatgatgacttcaacacaatgggctactgtgaggagatccccatagaagagaatgaagtgaatgacagctcatccaaaagtagca
tcgagaccaagccggatgccagcccacagctgcccaagaagtccattaccaacagcacactgacgtctacgggaagcagtgaagcccctgtttcgtttgatggcttgccattggaggaggaggtgatggaggggga
tggttccctagagaaggagcttgccattgacaacatcataggggagaagatcgagatcatggcgcctgtgacctccccttcgctggacttcaatgacaatgaggatatccccaccgagctcagtgattcttcagatac
ccacgatgaaggagaggtccaggccttctatgaggacctgagtggaagacaatatgtgaatgaggtcttcaacttcagcgtggacaaactctatgacctgctgttcaccaactcacccttcctgcgggatttcatgga
gcagcggcgtttctctgatatcatcttccatccatggaaaaaggaggagaatggaaaccagagtcgagtgattctctacactatcaccctcactaatcctctggctcccaaaacagccactgtcagggagacacaga
ccatgtacaaagcgagtcaggagagcgaatgctacgtgatagatgctgaagtcctcacccacgatgtgccgtaccatgactacttctacaccatcaatcgctacacactcaccagagtggcccggaacaagagcag
actcagggtctccacagagctccgctatcgaaaacagccctggggggtttgtgaaaacgttcatcgagaagaacttctggagtggactggaggactacttccgtcatttagagactgaactaaccaaaacagagagt
acctacttggctgagatacacaggcagtcacccaaggagaaggctagcaagtcttcagcagtccggaggaggaagcgtccccatgcccacctgcgggtgcctcacctggaagaagtgatgagtcctgtcaccaca
cccactgatgaagatgtgggccacaggatcaagcacgtggcaggttccacgcagacacggcacatcccggaggacactcctgatggttttcacctacaaagtgtgtccaagctgctgctggttatcagctgtgttctg
gtgctgctggttgtccttaacatgatgctcttctacaagctgtggatgctggaatacaccacccagaccctcactgcctggcagggtctcaggctccaggaaaggttaccccagtctcagacagaatgggcccagcta
ttagaatcccaacagaagtaccacgacaccgagctccagaagtggagggaaatcatcaaatcctcagtgttgctcctggaccagatgaaggactcgcttatcaatcttcagaatggtatcaggtcccgagactaca
cagctgagagcgacgagaagaggaaccgctatcattga

C

Mouse *Gramd1b2* Full length Sequence

Exon 1; Exon 2; Exon 3; Exon 4

GRAM domain; ASTER domain; TM

atgccggcggccaacatgatggagaacctgcagctgcccgccctgcaggtgccagaacctcagggcgcacccgagggcagcgcggtgtggtccagctcgtccacccccacgcttcgccgccggcgctttaagatgc
gccggatgaagaacgtgcaggagcagagcctggaggcggggttggtagccccggacctacccggcgtcctggcccccggcaaggagttcctgcaactgccgtccattgagattacgccctccagcgacgaggaca
ccccgtggtccaactgctccacacccagtgcgtccccgcgccgaaaacgcttcctgctccgcaagtggctgagggtgagggagcggaaagaatgcagtgaaagcagcagccagcaaagcagccaacagagcagc
cacgacgatgattcgagcaggttcctgagtcctcgagtgcgcgacgaaagcactgccagtaactccaaccgcagcacgccagcctgctctcccatcctccggaagcggtcccgctcaccaacaccacagaaccagg
acggagacaccatggtggagaagggctcagatcactcctcggacaagtccccgtccaccccggagcagggtgtgcaacgcagttgctcctcacagtctggtcggagcggtggtaaaaattccaagaaaagccaga
gttggtacaatgtgctaagccccacttacaagcagagaaatgaagacttcagaaagctctttaagcaacttccagacacggagcgcctcattgttgattactcttgtgcactccaaagagacattcttcttcagggcc
gactctacctctcagaaaattggatctgcttctacagcaacatcttccgctgggaaaccctgctgacagtccgtttgaaagatatctgctccatgactaaagagaaaacagctcgcctcattcccaatgccatccaagt
ctgcactgattcagaaaagcactttttacttcctttggagcccgggacaggacgtacatgatgatgttccggctctggcagaatgctctccttgaaaagcccctatgtcccaaggagctctggcactttgtccaccagt
gctacgggaacgagctgggcctaaccagtgatgatgaggactacgtgcctccagatgatgacttcaacacaatgggctactgtgaggagatccccatagaagagaatgaagtgaatgacagctcatccaaaagta
gcatcgagaccaagccggatgccagcccacagctgcccaagaagtccattaccaacagcacactgacgtctacgggaagcagtgaagcccctgtttcgtttgatggcttgccattggaggaggaggtgatggaggg
ggatggttccctagagaaggagcttgccattgacaacatcataggggagaagatcgagatcatggcgcctgtgacctccccttcgctggacttcaatgacaatgaggatatccccaccgagctcagtgattcttcag
atacccacgatgaaggagaggtccaggccttctatgaggacctgagtggaagacaatatgtgaatgaggtcttcaacttcagcgtggacaaactctatgacctgctgttcaccaactcacccttcctgcgggatttca
tggagcagcggcgtttctctgatatcatcttccatccatggaaaaaggaggagaatggaaaccagagtcgagtgattctctacactatcaccctcactaatcctctggctcccaaaacagccactgtcagggagaca
cagaccatgtacaaagcgagtcaggagagcgaatgctacgtgatagatgctgaagtcctcacccacgatgtgccgtaccatgactacttctacaccatcaatcgctacacactcaccagagtggcccggaacaaga
gcagactcagggtctccacagagctccgctatcgaaaacagccctggggggtttgtgaaaacgttcatcgagaagaacttctggagtggactggaggactacttccgtcatttagagactgaactaaccaaaacaga
gagtacctacttggctgagatacacaggcagtcacccaaggagaaggctagcaagtcttcagcagtccggaggaggaagcgtccccatgcccacctgcgggtgcctcacctggaagaagtgatgagtcctgtcacc
acacccactgatgaagatgtgggccacaggatcaagcacgtggcaggttccacgcagacacggcacatcccggaggacactcctgatggttttcacctacaaagtgtgtccaagctgctgctggttatcagctgtgt
tctggtgctgctggttgtccttaacatgatgctcttctacaagctgtggatgctggaatacaccacccagaccctcactgcctggcagggtctcaggctccaggaaaggttaccccagtctcagacagaatgggccca
gctattagaatcccaacagaagtaccacgacaccgagctccagaagtggagggaaatcatcaaatcctcagtgttgctcctggaccagatgaaggactcgcttatcaatcttcagaatggtatcaggtcccgagact
acacagctgagagcgacgagaagaggaaccgctatcattga

D

```
Aster-B1 ------------------------------------------------------------ 0
Aster-B2 MPAANMMENLQLPALQVPEPQGAPEGSAVWSSSSTPTLRRRRFKMRRMKNVQEQSLEAGL 60

Aster-B1 ------------------------------------------------------------ 0
Aster-B2 VAPDLPGVLAPGKEFLQLPSIEITPSSDEDTPWSNCSTPSASPRRKRFLLRKWLRVRERK 120

Aster-B1 ------------------------------------------------------------ 0
Aster-B2 ECSESSSQQSSQQSSHDDDSSRFLSPRVRDESTASNSNRSTPACSPILRKRSRSPTPQNQ 180

Aster-B1 -MESLTESGVLWSLLLELDSQSLLWYLKRLADAPVGAECYCW---------HGSEKIPAV 50
Aster-B2 DGDTMVEKGSDHSSD----------KSPSTPEQGVQRSCSSQSGRSGGKNSKKSQSWYNV 230
           :::.*.*   *                  :  *  .* .          : *:.   *

Aster-B1 LSPTYKQRNEDFRKLFKQLPDTERLIVDYSCALQRDILLQGRLYLSENWICFYSNIFRWE 110
Aster-B2 LSPTYKQRNEDFRKLFKQLPDTERLIVDYSCALQRDILLQGRLYLSENWICFYSNIFRWE 290
         ************************************************************
```

E

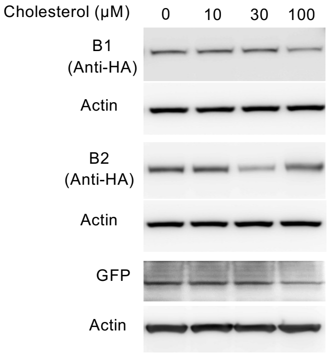

Xiao_Fig S2.

A

B

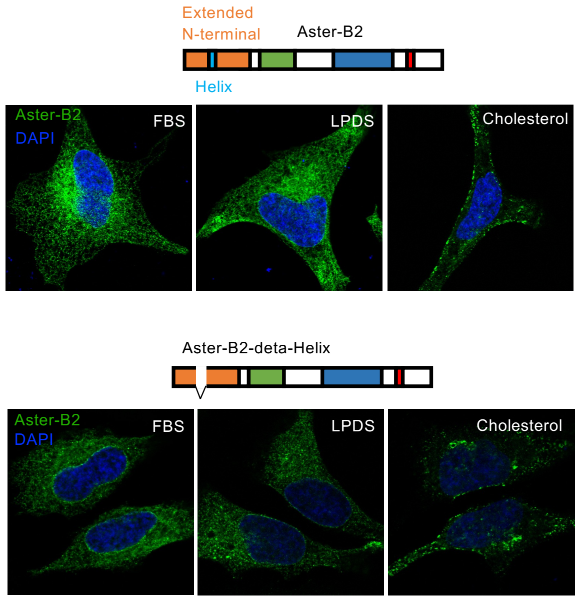

C

Aster-B1		
Amino acid composition:		
Leu (L)	10	20.40%
Ser (S)	6	12.20%
Glu (E)	5	10.20%
Ala (A)	4	8.20%
Gly (G)	3	6.10%
Trp (W)	3	6.10%
Asp (D)	2	4.10%
Cys (C)	2	4.10%
Tyr (Y)	2	4.10%
Val (V)	2	4.10%
Pro (P)	2	4.10%
Lys (K)	2	4.10%
Arg (R)	1	2.00%
Gln (Q)	1	2.00%
His (H)	1	2.00%
Ile (I)	1	2.00%
Met (M)	1	2.00%
Thr (T)	1	2.00%
Phe (F)	0	0.00%
Asn (N)	0	0.00%

Aster-B2		
Amino acid composition:		
Ser (S)	44	19.20%
Arg (R)	22	9.60%
Pro (P)	21	9.20%
Gln (Q)	16	7.00%
Leu (L)	15	6.60%
Glu (E)	14	6.10%
Lys (K)	12	5.20%
Asp (D)	11	4.80%
Gly (G)	11	4.80%
Ala (A)	11	4.80%
Thr (T)	10	4.40%
Val (V)	9	3.90%
Asn (N)	9	3.90%
Met (M)	6	2.60%
Cys (C)	4	1.70%
Trp (W)	4	1.70%
Phe (F)	4	1.70%
Ile (I)	3	1.30%
His (H)	2	0.90%
Tyr (Y)	1	0.40%

D E F

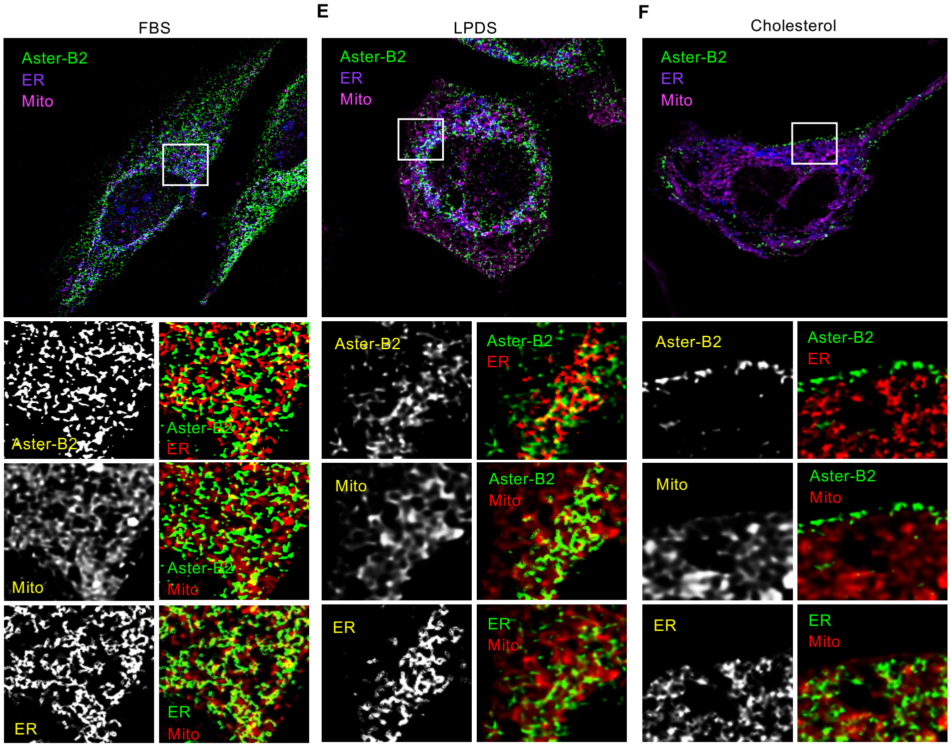

Xiao_Fig S3.

A

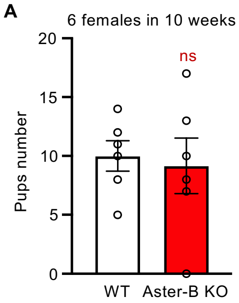

B

M x F	Total Pups NO.	Pups/mouse	Total Litters NO.	Litters/mouse
WT x WT	60	10	11	1.83
WT x KO	55	9.17	8	1.33

Xiao_Fig S4.

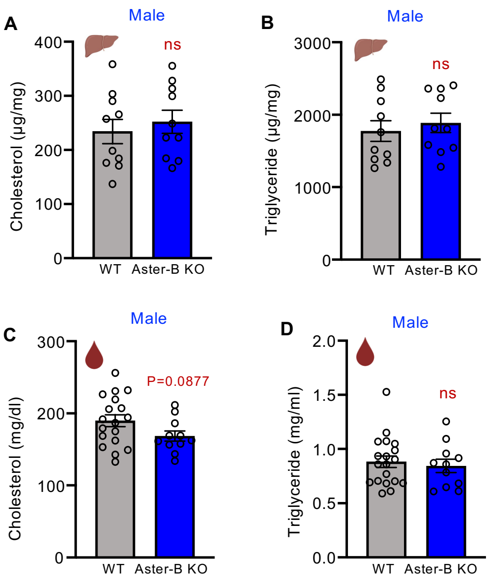

A
Male
400
300
200
100
0
Cholesterol (μg/mg)
ns
WT
Aster-B KO
B
Male
3000
2000
1000
0
Triglyceride (μg/mg)
ns
WT
Aster-B KO
C
Male
300
200
100
0
Cholesterol (mg/dl)
P=0.0877
WT
Aster-B KO
D
Male
2.0
1.5
1.0
0.5
0.0
Triglyceride (mg/ml)
ns
WT
Aster-B KO

Xiao_Fig S5.

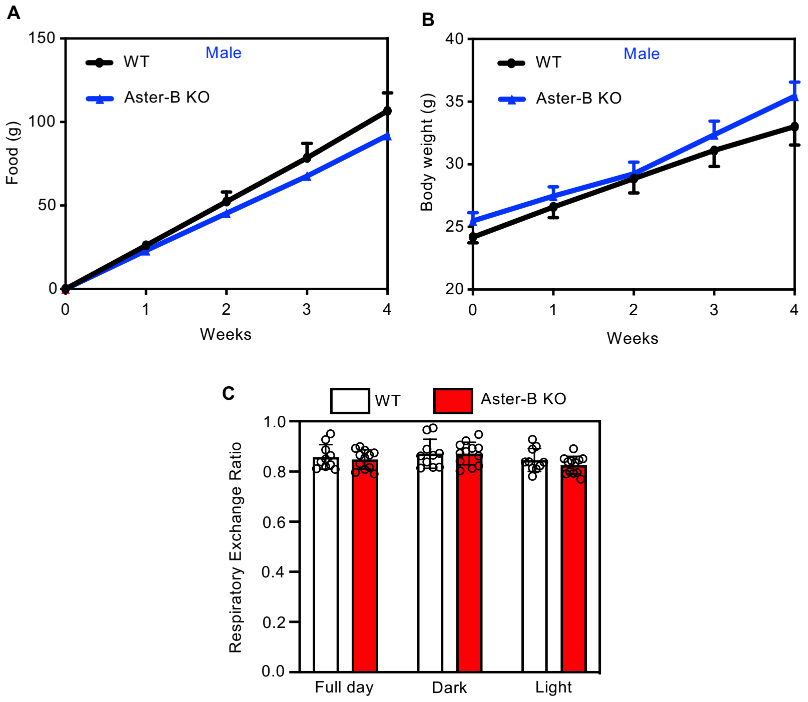
A
Food (g)
150
100
50
0
WT
Aster-B KO
Male
0
1
2
3
4
Weeks
B
Body weight (g)
40
35
30
25
20
WT
Aster-B KO
Male
0
1
2
3
4
Weeks
C
WT
Aster-B KO
Respiratory Exchange Ratio
1.0
0.8
0.6
0.4
0.2
0.0
Full day
Dark
Light

Xiao_Fig S6.

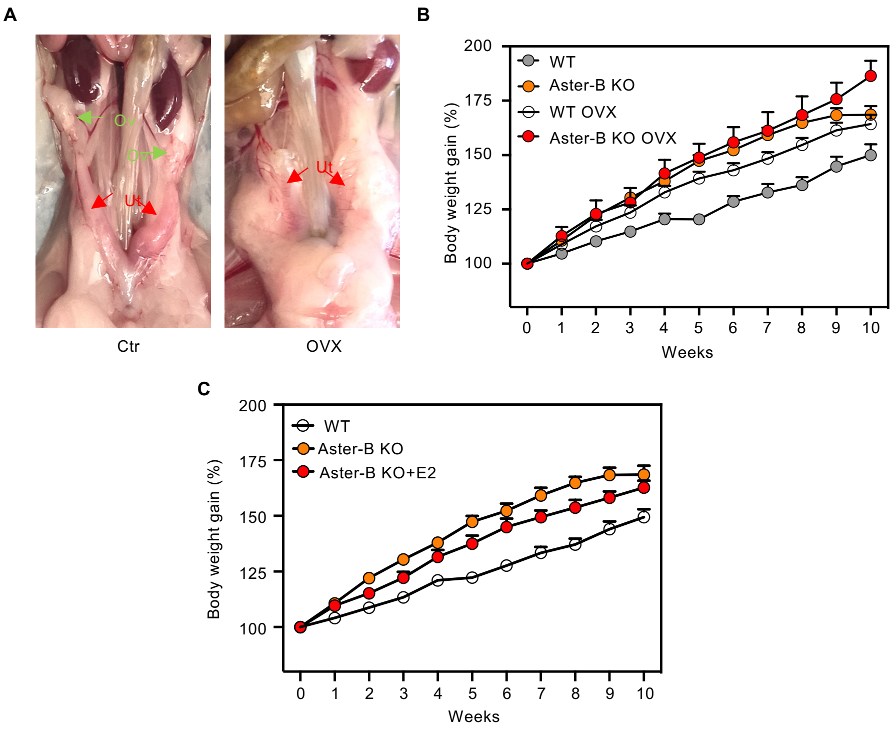
A
Ov
Ov
Ut
Ut
Ctr
OVX
B
WT
Aster-B KO
WT OVX
Aster-B KO OVX
Body weight gain (%)
200
175
150
125
100
0
1
2
3
4
5
6
7
8
9
10
Weeks
C
WT
Aster-B KO
Aster-B KO+E2
Body weight gain (%)
200
175
150
125
100
0
1
2
3
4
5
6
7
8
9
10
Weeks

Xiao_Fig S7.

Supplemental Table 1. Q-PCR primer sequences

Gene	Forward	Reverse
m Gramd1A	CAGCAGATGCTCTTCTCGGA	TCTGAGGATACACGAAGCCG
m Gramd1b	TCCCAATGCCATCCAAGTC	ACAAAGTGCCAGAGCTCC
m Gramd1c	CCGTGTCTTTCACATCAGTGC	ACTTCCCAGTTAGCGGGTTG
m Srebp2	ACCTAGACCTCGCCAAAGGT	GCACGGATAAGCAGGTTTGT
m Hmgcr	CTTGTGGAATGCCTTGTGATT	AGCCGAAGCAGCACATGAT
m Hmgcs	GCC GTG AAC TGG GTC GAA	GCA TAT ATA GCA ATG TCT CCT
m Ldlr	AGGCTGTGGGCTCCATAGG	TGC GGT CCA GGG TCA TCT
m Sqle	GCCTCTCAGAATGGTCGTCT	CGCATGTCCCAGAATAAGGA
m Gdf9	GTCACCTCTACAATACCGTCCG	TAAACAGCAGGTCCACCATCGG
m Bmp15	GATTGGAGCGAAAATGGTGAGGC	GCTACCTGGTTTGATGCTAGAGG
m Caspase-3	GGAGTCTGACTGGAAAGCCGAA	CTTCTGGCAAGCCATCTCCTCA
m Bcl2	CCTGTGGATGACTGAGTACCTG	AGCCAGGAGAAATCAAACAGAG
m Bcl3	AGCAGTCGTCTCAGCTCCAATG	AGGCAGGTGTAGATGTTGTGGG
m p21	TCGCTGTCTTGCACTCTGGTGT	CCAATCTGCGCTTGGAGTGATAG
m p53	CTGGTTAGTCCTGAGACAGAGG	AGATGCAGCCAAACACAGGCAC
m Bax	AGGATGCGTCCACCAAGAAGCT	TCCGTGTCCACGTCAGCAATCA
m Esr1	GCCAGAATGGCCGAGAGAG	CCCCATAATGGTAGCCAGAGG
m Pgr	CTACTCGCTGTGCCTTACCATG	CTGGCTTTGACTCCTCAGTCCT
m Pck1	GGCGATGACATTGCCTGGATGA	TGTCTTCACTGAGGTGCCAGGA
m Gdf15	AGCCGAGAGGACTCGAACTCAG	GGTTGACGCGGAGTAGCAGCT
m Polg1	TAGCTGGCTGGTCCAAGAGT	CGACGTGGAGGTCTGCTT
m Nrf1	GACAAGATCATCAACCTGCCTGTAG	GCTCACTTCCTCCGGTCCTTTG
m Fasn	AAGTCCCAGAAATCGCCTATG	GGTATGGTTTCACGACTGGAG
m Acc	GTTCTGTTGGACAACGCCTTCAC	GGAGTCACAGAAGCAGCCCATT
m Scd1	TTCTTGCGATACACTCTGGTGC	CGGGATTGAATGTTCTTGTCGT
m Elovl6	CGGCATCTGATGAACAAGCGAG	GTACAGCATGTAAGCACCAGTTC
m Pgc1a	TATGGAGTGACATAGAGTGTGCT	GTCGCTACACCACTTCAATCC
m Ucp1	ACTGCCACACCTCCAGTCATT	CTTTGCCTCACTCAGGATTGG
m Adrb3	AGAAACGGCTCTCTGGCTTTG	TGGTTATGGTCTGTAGTCTCGG
m Cox8b	GAACCATGAAGCCAACGACT	GCGAAGTTCACAGTGGTTCC
m Cox4i1	ATTGGCAAGAGAGCCATTTCTAC	TGGGGAAAGCATAGTCTTCACT
m Cox5b	GCTGCATCTGTGAAGAGGACAAC	CAGCTTGTAATGGGTTCCACAGT
m Cox7a1	CAGCGTCATGGTCAGTCTGT	AGAAAACCGTGTGGCAGAGA
