# Supplementary material for: Aster-B–dependent estradiol synthesis protects female mice from diet-induced obesity
Source: J Clin Invest. 2024 Jan 4;134(4):e173002. doi: 10.1172/JCI173002 (PMC10866650; doi:10.1172/JCI173002)

Full unedited gel for Fig.S2E

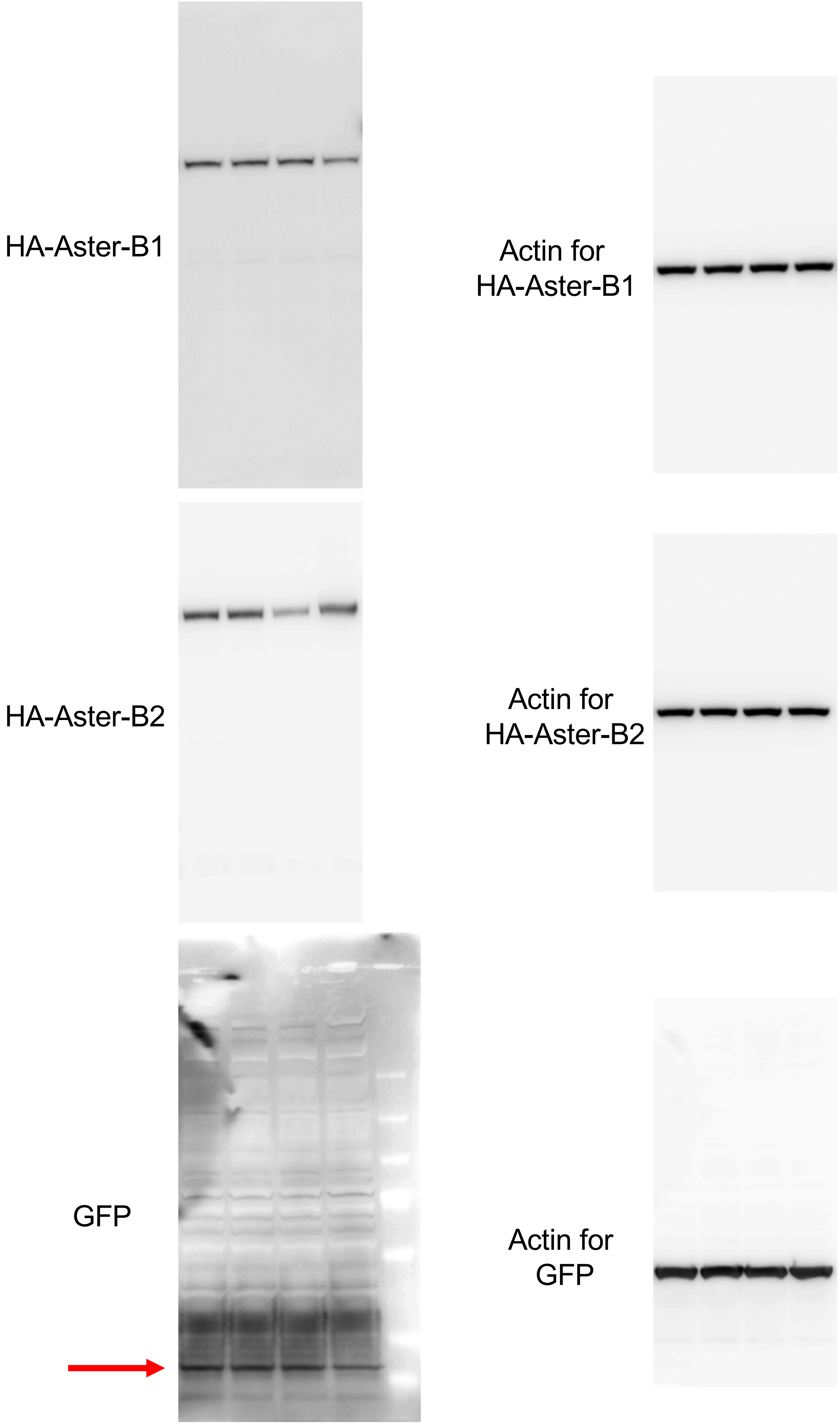

Supplement: Unedited blot and gel images [file jci-134-173002-s112.pdf]
